# Functional consequence of pathogenic *GABRA3* variants determines whether X-linked inheritance is dominant or recessive

**DOI:** 10.1172/JCI189830

**Published:** 2025-11-25

**Authors:** Katrine M. Johannesen, Khaing Phyu Aung, Vivian W.Y. Liao, Nathan Absalom, Han C. Chua, Xue N. Gan, Miaomiao Mao, Chaseley E. McKenzie, Hian M. Lee, Sebastian Ortiz, Rebecca C. Spillmann, Vandana Shashi, Rodney A. Radtke, Ghayda M. Mirzaa, P. Anne Weisner, Josue Flores Daboub, Caroline Hagedorn, Pinar Bayrak-Toydemir, Desiree DeMille, Jian Zhao, Nandita Bajaj, Yline Capri, Boris Keren, Miriam Schmidts, Ingrid M.B.H. van de Laar, Marjon A. van Slegtenhorst, Rafal Ploski, Marta Bogotko, Danielle K. Bourque, Ebba Alkhunaizi, Lauren Chad, Nada Quercia, Houda Elloumi, Ingrid M. Wentzensen, Michael C. Kruer, Pritha Bisarad, Carolina I. Galaz-Montoya, Violeta Rusu, Dominique Braun, Katie Angione, Jessica C. Win, Camilo Espinosa-Jovel, Pia Zacher, Konrad Platzer, Samuel F. Berkovic, Ingrid E. Scheffer, Mary Chebib, Guido Rubboli, Rikke S. Møller, Christopher A. Reid, Philip K. Ahring

**Affiliations:** 1 Department of Epilepsy Genetics and Personalized Treatment, Danish Epilepsy Centre, Filadelfia, Dianalund, Denmark.; 2 Department of Genetics, University Hospital of Copenhagen, Rigshospitalet, Copenhagen, Denmark.; 3 The Florey Institute of Neuroscience and Mental Health, University of Melbourne, Parkville, Victoria, Australia.; 4 School of Medical Sciences, Faculty of Medicine and Health, Brain and Mind Centre, The University of Sydney, Camperdown, New South Wales, Australia.; 5 School of Science, The MARCS Institute for Brain, Behaviour and Development, University of Western Sydney, Sydney, New South Wales, Australia.; 6 Sydney Pharmacy School, Faculty of Medicine and Health and Charles Perkins Centre, The University of Sydney, Sydney, New South Wales, Australia.; 7 Department of Regional Health Research, Faculty of Health Sciences, University of Southern Denmark, Odense, Denmark.; 8 Department of Pediatrics, Division of Medical Genetics, and; 9 Department of Neurology, Duke University School of Medicine, Durham, North Carolina, USA.; 10 Department of Laboratory Medicine and Pathology and; 11 Department of Pediatrics, University of Washington School of Medicine, Seattle, Washington, USA.; 12 Brotman Baty Institute for Precision Medicine, Seattle, Washington, USA.; 13 Department of Pediatrics, Genetics Division, University of Utah School of Medicine, Salt Lake City, Utah, USA.; 14 Department of Pathology, University of Utah, Salt Lake City, Utah, USA.; 15 ARUP Institute for Clinical and Experimental Pathology, Salt Lake City, Utah, USA.; 16 Department of Neuropediatrics, Christian Children’s Hospital Osnabrück, Osnabrück, Germany.; 17 Department of Genetics, Robert Debré Hospital, Assistance Publique–Hôpitaux de Paris Nord, University of Paris, Paris, France.; 18 Department of Medical Genetics, Pitié-Salpêtrière Hospital, Assistance Publique–Hôpitaux de Paris, Sorbonne Université, Paris, France.; 19 Center for Pediatrics and Adolescent Medicine, Freiburg University Hospital, Faculty of Medicine, and; 20 CIBSS – Centre for Integrative Biological Signaling Studies, University of Freiburg, Freiburg, Germany.; 21 Department of Clinical Genetics, Erasmus University Medical Center, Rotterdam, Netherlands.; 22 Department of Medical Genetics, Warsaw Medical University, Warsaw, Poland.; 23 Professor Jan Bogdanowicz Children’s Hospital, Warsaw, Poland.; 24 Division of Metabolics and Newborn Screening, Department of Pediatrics, Children’s Hospital of Eastern Ontario, University of Ottawa, Ottawa, Canada.; 25 Division of Clinical and Metabolic Genetics, Department of Pediatrics, The Hospital for Sick Children, University of Toronto, Toronto, Canada.; 26 GeneDx, Gaithersburg, Maryland, USA.; 27 Pediatric Movement Disorders Program, Division of Pediatric Neurology, Barrow Neurological Institute, Phoenix Children’s Hospital, Phoenix, Arizona, USA.; 28 Departments of Child Health, Neurology, and Cellular and Molecular Medicine, and Program in Genetics, The University of Arizona College of Medicine–Phoenix, Phoenix, Arizona, USA.; 29 Genetics, Graduate Interdisciplinary Program, The University of Arizona, Tucson, Arizona, USA.; 30 Department of Human Genetics, Inselspital, Bern University Hospital, University of Bern, Bern, Switzerland.; 31 Department of Child Neurology, Children’s Hospital Colorado, Aurora, Colorado, USA.; 32 School of Medicine, University of Colorado Denver, Aurora, Colorado, USA.; 33 Department of Neurology, Riley Children’s Health, Indiana University School of Medicine, Indianapolis, Indiana, USA.; 34 Epilepsy Program, Instituto Roosevelt, Bogotá, Colombia.; 35 Center for Adults with Disability (MZEB), Epilepsy Center Kleinwachau, Radeberg, Germany.; 36 Institute of Human Genetics, University of Leipzig Medical Center, Leipzig, Germany.; 37 Department of Medicine, Epilepsy Research Centre, University of Melbourne, Austin Health, Heidelberg, Victoria, Australia.; 38 Royal Children’s Hospital, Florey Institute and Murdoch Children’s Research Institute, Melbourne, Victoria, Australia.; 39 Institute of Clinical Medicine, University of Copenhagen, Copenhagen, Denmark.

**Keywords:** Genetics, Neuroscience, Epilepsy, Genetic diseases, Intellectual disability

## Abstract

Disorders of *GABRA3*, the only epilepsy-associated GABA_A_ receptor subunit gene on the X chromosome, have eluded clinical clarity due to ambiguous inheritance patterns and variable phenotypes. The long-standing assumption that all pathogenic variants cause loss of function further obscured genotype–phenotype relationships and hindered progress. Here, we curated a cohort of individuals with a *GABRA3* variant, integrating deep phenotyping, genotyping, family history, and electrophysiology with a targeted mouse model. Among 43 individuals with 19 *GABRA3* variants, functional analyses revealed gain- and loss-of-function effects, each linked to distinct clinical profiles. Gain-of-function variants were associated with severe, treatment-resistant epilepsy and profound intellectual disability, disproportionately affecting males, who were often nonambulant and had cortical visual impairment. Loss-of-function variants produced milder phenotypes, with epilepsy rarely observed; affected males showed behavioral issues and language delay, while females were unaffected carriers. Our gain-of-function (*Gabra3*^Q242L/+^) mouse model mirrored these sex-specific differences, showing increased seizure susceptibility, early death, and marked cortical hyperexcitability. These insights resolve longstanding uncertainties surrounding *GABRA3* and redefine how X-linked disorders are interpreted. They demonstrate that it is the functional impact of a variant, not its mere presence, that determines whether a condition manifests dominantly or recessively. This distinction carries important implications for genetic counseling, precision medicine, and the broader interpretation of X-linked neurodevelopmental disorders.

## Abstract 2

Disease-causing variants in the GABRA3 gene, located on the X-chromosome, causes neurodevelopmental disorders, where severity and type depends on the functional effect of the variant

## Graphical abstract

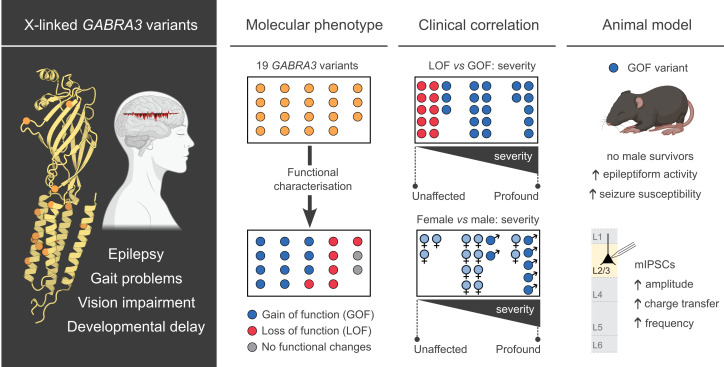

## Introduction

GABA type A (GABA_A_) receptors are ligand-gated ion channels that mediate fast synaptic inhibition in the brain (1). Among the 19 identified subunits (α1–6, β1–3, γ1–3, δ, ε, θ, π, and ρ1–3), the α3 subunit, encoded by *GABRA3*, stands out for several reasons. First, *GABRA3* is the only epilepsy-associated GABA_A_ receptor gene that is located on the X chromosome. Second, compared with more common receptor types, α3-containing receptors exhibit restricted spatial expression in the brain, with high levels in regions important for epilepsy, such as the cerebral cortex, amygdala, hippocampus, and thalamus (2). Third, α3 subunit–containing receptors mediate synaptic neurotransmission with prolonged synaptic currents (3). Together, these unique features separate α3-containing receptors from, e.g., the more ubiquitous α1-containing receptors.

Despite its recognition as an epilepsy-associated gene, many aspects of *GABRA3* remain obscure. In the first report by Niturad et al. (4), affected individuals had a strikingly diverse range of phenotypes across both sexes, including severe developmental and epileptic encephalopathies (DEEs), intellectual disability (ID) with or without seizures, and, in some cases, no symptoms at all. One family showed a pattern consistent with X-linked dominant inheritance, yet findings in other families suggested that variation in *GABRA3* may act more as a genetic risk factor than a monogenic cause due to the presence of phenocopies and unaffected male carriers. These patterns therefore do not align with classical X-linked inheritance. Furthermore, functional analyses of these presumed pathogenic *GABRA3* variants identified loss of function (LOF) as the only pathogenic mechanism (3, 4). However, this cannot fully account for the wide range of clinical phenotypes, nor does it reconcile with the mild phenotype of *Gabra3*-KO mice (5). In stark contrast, recent genotype–phenotype correlations for other GABA_A_ receptor genes, including *GABRA1* (6), *GABRB1-3* (7–10), *GABRD* (11), and *GABRG2* (12), indicate that gain-of-function (GOF) variants are typically associated with more severe, treatment-resistant epilepsy, whereas LOF variants tend to result in milder forms. It is currently unclear if these rules apply to *GABRA3*, given its X-linked nature.

In this study, we assembled a cohort of 43 individuals with presumed pathogenic *GABRA3* variants and conducted detailed genotype–phenotype analyses. Our findings revealed that pathogenic *GABRA3* variants can cause either dominant or recessive X-linked disorders, each associated with distinct functional impact: GOF variants caused severe phenotypes and followed X-linked dominant inheritance, while LOF variants resulted in milder phenotypes and followed an X-linked recessive pattern. Key aspects of the human condition, including epilepsy and increased severity in males, were recapitulated in a mouse model carrying a GOF variant. These insights deepen our understanding of *GABRA3*-related disease and underscore how functional outcomes affect disease penetrance for X-linked disorders, demonstrating that GOF GABA_A_ receptor disease can be effectively modeled in animals.

## Results

**Figure 1:**
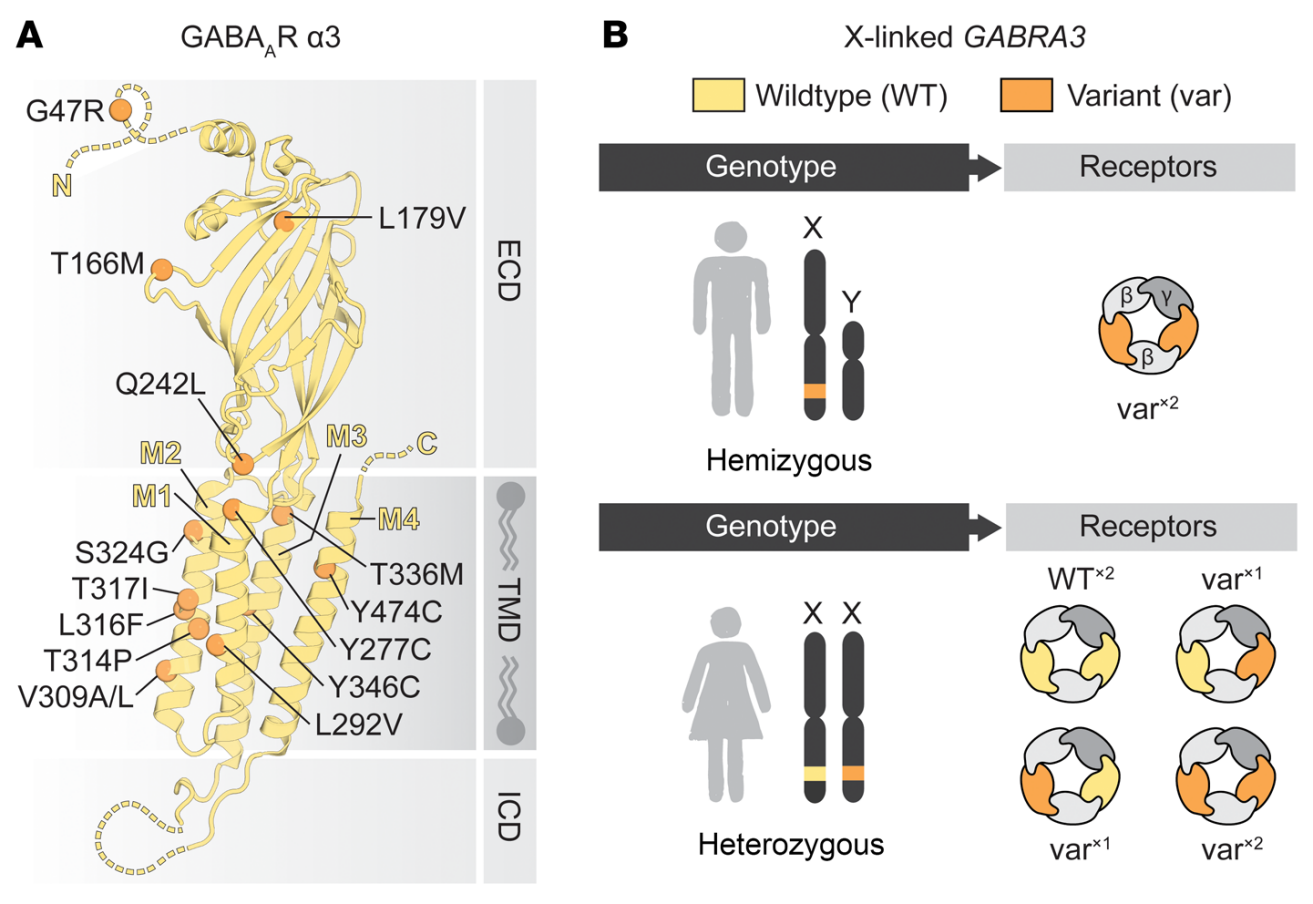
Presumed pathogenic variants of the X-linked *GABRA3* gene. (**A**) Structural mapping of the 15 missense variants reported in this study on the native mouse GABA_A_ receptor α3 structure (PDB 8G4X). ECD, extracellular domain; TMD, transmembrane domain; ICD, intracellular domain. (**B**) The *GABRA3* gene is located on the human X chromosome, which has a direct influence on the GABA_A_ receptor populations that different variant carriers express.

The cohort consisted of 43 individuals, 18 males and 25 females, carrying missense or protein-truncating *GABRA3* variants (Supplemental Table 1; supplemental material available online with this article; https://doi.org/10.1172/JCI189830DS1). Twenty-four individuals were previously unreported and included 22 probands. Nineteen distinct *GABRA3* variants were identified: 15 missense variants in 36 individuals, and 4 protein-truncating variants in 7 individuals. Protein-truncating variants have not been described for *GABRA3* before and can be assumed to cause LOF and are likely pathogenic. Most missense variants are located in regions critical for channel function, with 11 found in the transmembrane domain helices, which participate in forming the ion channel (Figure 1A). One variant, p.(Gln242Leu), is in the coupling region (loop 9), and another, p.(Leu179Val), is near the GABA binding site. The remaining 2 variants, p.(Gly47Arg) and p.(Thr166Met), were not in regions of known specific importance. Because *GABRA3* is X linked, males express only GABA_A_ receptors containing 2 variant α3 subunits, while females produce a mix of receptors: those with 2 variant subunits, 1 variant and 1 WT, and 2 WT subunits (Figure 1B). Assuming a binomial distribution, these populations would occur at approximately 25%, 50%, and 25%, respectively. Although factors like X inactivation can influence these ratios, prior studies suggest minimal impact (4), supporting the binomial model. As a result, receptors with a single variant α3 subunit are expected to predominate in females. To assess potential sex-specific effects, it is therefore critical to evaluate receptors with 1 and 2 variant α3 subunits alongside those with only WT subunits.

### Functional analysis reveals primarily GOF effects.

For electrophysiological analysis, α3* subunits carrying point mutations were studied in α3β3γ2 assemblies. To ensure consistent expression of defined receptor populations in *Xenopus laevis* oocytes, pentameric concatenated constructs were engineered to include either 2 WT, 1 WT and 1 mutant, or 2 mutant α3* subunits (13, 14). Functional analysis was performed using two-electrode voltage clamp electrophysiology, and 2 receptor parameters were evaluated: the sensitivity to GABA and the maximal GABA-evoked current amplitudes.

**Figure 2:**
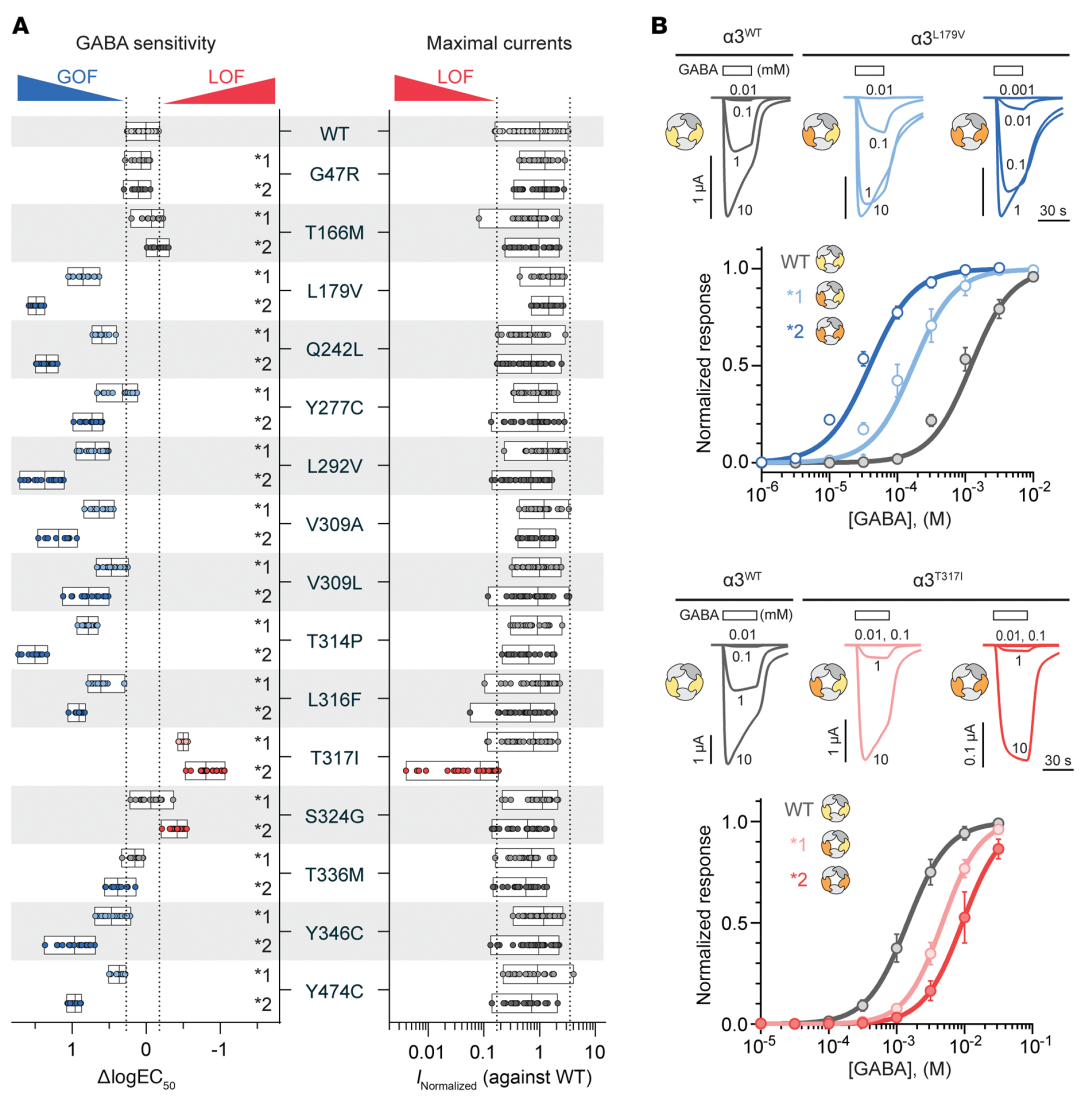
Functional impact of α3 subunit mutations on GABA sensitivity and current amplitude in α3β3γ2 GABA_A_ receptors. WT and 30 mutant α3-containing receptors were expressed in *Xenopus laevis* oocytes and analyzed via two-electrode voltage clamp electrophysiology. (**A**) Changes in GABA sensitivity between the receptors with α3 subunit mutations and the WT were calculated from the logarithmic conversion of EC_50_ values (ΔlogEC_50_) on each experimental day. Final ΔlogEC_50_ datasets for each mutant receptor contain data from *n* = 9–18 independent experiments and are presented as individual data points, with floating bars indicating the range and a line denoting the mean. One-way ANOVA [*F*(30, 519) = 430; *P* < 0.0001] with Dunnett’s post hoc test identified mutations with significantly increased (blue) or decreased (red) sensitivity (*P* < 0.0001); nonsignificant changes are shown in gray. Maximal GABA-evoked current amplitudes were normalized and log transformed. Final datasets included *n* = 23–36 experiments per variant, presented as individual data points, with floating bars indicating the range and a line denoting the mean. Mann-Whitney *U* tests compared each mutant with WT controls from matched experimental days. Mutations with reduced peak currents (*P* < 0.0001) are shown in red. (**B**) Representative current traces for WT, p.(Leu179Val), and p.(Thr317Ile) receptors illustrate responses to increasing GABA concentrations. Corresponding concentration–response relationships for *n* = 13–16 experiments are plotted as mean ± SD. The Hill equation was fitted to each dataset by nonlinear regression. Full electrophysiological details and regression parameters are provided in Supplemental Table 1.

All receptor combinations with mutant α3* subunits responded to GABA applications (Figure 2A). For 10 mutations, α3^L179V^, α3^Q242L^, α3^Y277C^, α3^L292V^, α3^V309A^, α3^V309L^, α3^T314P^, α3^L316F^, α3^Y346C^, and α3^Y474C^, both single- and double-mutant receptors showed marked increases in GABA sensitivity, with double-mutant receptors generally exhibiting 2-fold larger ΔlogEC_50_ values. None of the 10 mutations altered maximal GABA-evoked current amplitudes relative to the WT receptor. Thus, clear GOF traits were evident, with the α3^L179V^ mutation causing the largest shifts of 0.85 and 1.48 in ΔlogEC_50_ values, corresponding to 7.1- and 30-fold increases in GABA sensitivity, respectively (Figure 2B). In contrast, receptors containing the α3^T317I^ mutation showed reduced GABA sensitivity, particularly in the double-mutant receptor combination. This mutation additionally reduced the current amplitude in the double-mutant receptor, resulting in only 10% of the WT current remaining. The α3^T317I^ mutation thus resulted in clear LOF traits. Receptors with the α3^S324G^ and α3^T336M^ mutations showed intermediate functional changes. Single-mutant receptors displayed no significant alterations, whereas double-mutant receptors exhibited substantial shifts in GABA sensitivity. With a ΔlogEC_50_ value of –0.42, the double-mutant α3^S324G^ receptor displayed LOF traits; in contrast, the double-mutant α3^T336M^ receptor displayed GOF traits with a ΔlogEC_50_ value of 0.38 (Figure 2A). Finally, receptors containing the α3^G47R^ and α3^T166M^ mutations showed no notable differences in GABA sensitivity or maximal current amplitudes compared with the WT receptor, whether in single- or double-mutant configurations. These findings suggest that neither mutation impairs receptor function under the tested conditions.

### Reclassification of variants using ACMG guidelines.

Following functional analysis, missense variants were reclassified using American College of Medical Genetics and Genomics (ACMG) guidelines with pathogenic or benign support (PS3/BS3) codes based on a well-established assay (15). Twelve missense variants all displayed GOF or LOF traits in the functional analysis (PS3). Of these, 10 occurred de novo, with p.(Leu316Phe), p.(Tyr346Cys), and p.(Tyr474Cys) occurring both de novo and maternally inherited, and these can be classified as likely pathogenic or pathogenic depending on supporting phenotypic correlation. The remaining 2 variants were maternally inherited in severely affected males and are also classified as likely pathogenic (Supplemental Table 1).

For the p.(Ser324Gly) and p.(Thr336Met) variants, LOF and GOF traits were observed only in double-mutant receptors (PS3). Ser324 is located in the transmembrane M2 helix, Thr336 is in the M2-M3 coupling loop (Figure 1A), and both regions are important for receptor function (PM1). Based on functional data, these variants are likely detrimental in males, while they might not affect females. Considering all available information, both variants were classified as likely pathogenic (Supplemental Table 1). The p.(Ser324Gly) variant, observed in both sexes, is included in the genotype–phenotype analyses. Conversely, p.(Thr336Met) was found in a female proband and her mother (20a and 20b). The proband had bilateral tonic-clonic seizures with an EEG showing spike-and-wave complexes, indicative of genetic generalized epilepsy, while her mother was unaffected (4). Interestingly, the proband’s affected sister does not carry the *GABRA3* variant, and their father also experienced seizures. Thus, additional etiologies must be present in this family. As the proband’s phenotype cannot be attributed to the *GABRA3* variant, these 2 individuals were excluded from further genotype–phenotype analyses.

Finally, for the inherited p.(Gly47Arg) and p.(Thr166Met) variants, no noticeable changes were observed on functional testing (BS3). These variants are outside critical regions for receptor function and are each found in 6 entries in The Genome Aggregation Database, including hemizygous carriers. Thus, these 2 inherited variants likely represent benign polymorphisms (Supplemental Table 1). Consequently, the 11 individuals with these variants were excluded from our genotype–phenotype correlation analyses.

### Genotype–phenotype correlation underscores the severity of GOF variants.

**Figure 3:**
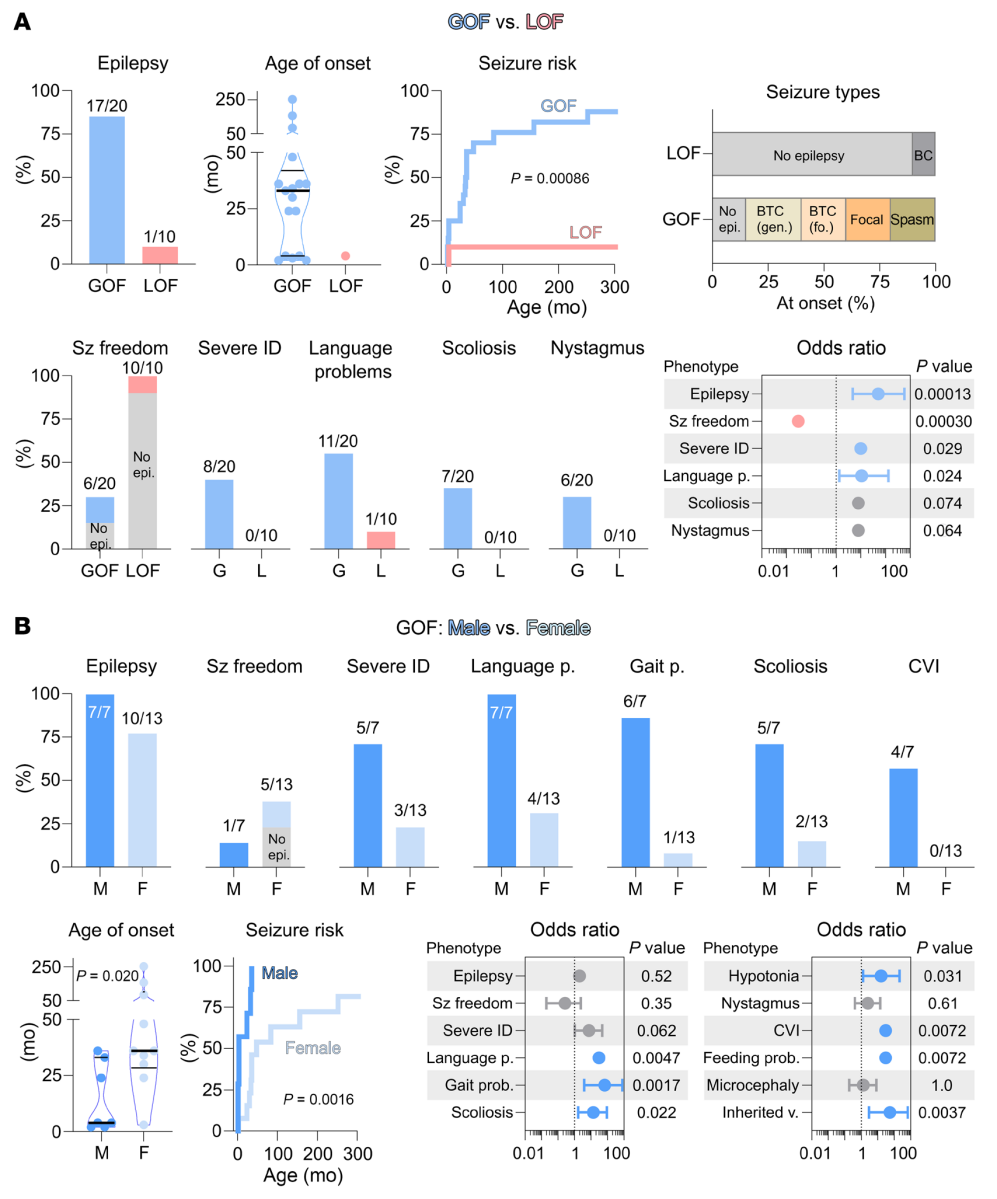
Clinical phenotypes of individuals with LOF and GOF *GABRA3* variants. Clinical parameters were evaluated to determine their correlation with the molecular characteristics of the variants or their connection to sex. (**A**) Comparison of individuals with GOF (*n* = 20) versus LOF (*n* = 10) variants. (**B**) Comparison of males (*n* = 7) versus females (*n* = 13) among individuals with GOF variants. Statistical analyses for seizure onset and seizure risk were performed using Mann-Whitney and Mantel-Cox tests, respectively, with the indicated *P* values. Seizure types depict only types at onset and include bilateral generalized clonic (BC), bilateral tonic-clonic (BTC) with either generalized (gen) or focal (fo) onset, focal tonic or impaired awareness or focal motor (Focal), and epileptic spasms (Spasm). OR analyses of phenotype–genotype associations are presented, with the center circle denoting the OR and error bars representing the 95% CI. Light and dark blue circles indicate significant enrichment in individuals with GOF (**A**) or males (**B**), respectively. Red circles indicate significant enrichment in individuals with LOF variants, and gray circles indicate no significant difference between compared individuals. Circles without CIs indicate data where 1 category contains either 0 or 100% of individuals and the OR and CI cannot be determined. Statistical analyses were performed using 2-sided Fisher’s exact test, resulting in the indicated *P* values. mo, age in months.

Among the 20 individuals with GOF variants (Table 1), 85% (17/20) had epilepsy with a median age of onset at 33 months (range 2–252 months) (Figure 3A). In contrast, only 10% (1/10) with LOF variants had epilepsy with onset at 4 months. Thus, the risk of developing seizures was higher for individuals with GOF variants (Mantel-Cox test, χ^2^ = 11; *P* = 0.00086) (17/20 GOF vs. 1/10 LOF, OR = 51 [95% CI: 4.7–580], Fisher’s exact test; *P* = 0.00013). In the GOF group, common seizure types at onset included bilateral tonic-clonic seizures with either focal or generalized onset, focal seizures, and epileptic spasms. Seizure freedom was achieved in only 18% (3/17) of affected individuals in the GOF group, as well as in the single individual with seizures in the LOF group. Consequently, the likelihood of living seizure free was lower for the GOF group (6/20 vs. 10/10, OR = not determined [ND]; *P* = 0.00030).

Additionally, individuals with GOF variants were more likely to have severe ID (8/20 vs. 0/10, OR = ND; *P* = 0.029) and severe language impairment (11/20 vs. 1/10, OR = 11 [1.4–130]; *P* = 0.019) (Figure 3A). Other factors such as scoliosis (7/20 vs. 0/10, OR = ND; *P* = 0.064), nystagmus (6/20 vs. 0/10, OR = ND; *P* = 0.074), and microcephaly (5/20 vs. 0/10, OR = ND; *P* = 0.14) were more prevalent in the GOF group, but less frequent. No difference was seen for gait problems (7/19 vs. 2/9, OR = 2.0 [0.32–12]; *P* = 0.67). While this highlights some of the variations between the GOF and LOF groups, even larger differences were observed between males and females. The detailed phenotypic descriptions below categorize the phenotypic spectra by both variant types according to sex.

### GOF variants in males.

Seven individuals with GOF variants were male (2a, 2c, 5, 8a, 9a, 9b, and 12a) (Table 1). Variants were maternally inherited, except for a de novo mosaic variant (5; 80% variant/20% WT). All had epilepsy with a median onset age of 4 months (range 2–36 months) (Figure 3B). Seizure types at onset included epileptic spasms (4/7), focal tonic seizures (2/7), and bilateral tonic-clonic seizures (1/7). All individuals had abnormal EEG findings, including EEG with generalized spike-and-wave, multifocal, and unifocal epileptiform discharges. Individual 5, with a mosaic variant, was seizure free, while the remaining 6 had ongoing seizures (daily to weekly) at ages 4 to 34 years. All individuals had ID; 5 had severe ID, and 2 had moderate ID (2c and 8a), classifying them as likely having DEE. Language development was impaired in all 7 cases, with 5 cases being nonverbal.

Neurological findings included scoliosis (5/7), hypotonia (4/7), and nystagmus (3/7). Cortical visual impairment (CVI) was common (4/7), as were sleep disturbances (4/7) and feeding difficulties (4/7), with 2 requiring tube feeding (9a and 9b). Dysmorphic features included cleft palate (4/7) and micrognathia (4/7). MRI data were available for 3 individuals; 2 had normal MRIs (5 and 12a), and 1 had cerebellar vermis atrophy (9a). One individual was deceased (2c), but details of age and cause of death were not available.

### GOF variants in females.

Thirteen individuals with GOF variants were female (1, 2b, 2d, 3, 4, 6, 7, 8b, 9c, 10, 11, 12b, and 13), and of these, 2 were unaffected carriers (8b and 9c) (Table 1). Variants were de novo in 9 and maternally inherited in 1, and inheritance was unknown in 3 (Figure 3B). One individual was mosaic (9c), with 90% WT allele detected in blood. Ten individuals had epilepsy with a median age of onset of 36 months (range 3–252 months). Seizure types at onset were (focal to) bilateral tonic-clonic seizures in 8/10 and focal seizures in 2/10. EEGs, where available, were abnormal in all with epilepsy, showing multifocal or generalized spike-and-wave patterns (4/7). Seven had ongoing seizures (1, 2b, 3, 6, 7, 9, 10, and 13), and 2 individuals were seizure free (2d and 12b) on antiseizure medication (lamotrigine and levetiracetam) at ages 42 and 38 years, respectively. Seizure offset was unknown (2d) and 23 years (12b). Three did not have seizures; 1 had the mosaic variant (9c), possibly contributing to a milder phenotype; and another (individual 4) was only 4.5 years old at inclusion and might still develop seizures.

Neurological findings included nystagmus (3/13) and strabismus (3/13). Four were nonverbal, while 2 had delayed language development. Neuropsychiatric features were reported in 3 (anxiety or autism spectrum disorder [ASD]). Dysmorphic features were noted in 6, including retrognathia (2/6), microcephaly (2/6), and cleft palate (1/6). MRI data were available for 3; 1 was normal, 1 had delayed myelination, and 1 had a thin corpus callosum.

### LOF variants in males and females.

Ten individuals carried a LOF variant (Table 1), 6 were male probands (14a, 15, 16, 17a, 18a, and 19a), and 4 were female carriers (mothers). All 4 mothers (14b, 17b, 18b, and 19b) were unaffected. The variants included 4 protein truncating and 2 missense variants. In probands, 1 variant occurred de novo, while 4 were maternally inherited and 1 had unknown inheritance. Among the 6 males, one (18a) had epilepsy with seizure onset with bilateral tonic-clonic seizures at 4 months of age. The remaining 5 males did not have epilepsy. Cognitive abilities ranged from normal (1/6) to mild (3/6) to moderate ID (2/6). Language impairment was common (5/6), with rarer features including sleep and gastrointestinal problems (esophageal reflux or constipation). Two individuals (13a and 14) had ASD, with 1 also having attention-deficit/hyperactivity disorder (ADHD) (14a). Dysmorphic features were present in 3 individuals (14a, 15, and 16), including mandibular prognathia, strabismus, posteriorly rotated ears, pectus excavatum, and a cleft palate/lip. An MRI in 1 individual revealed abnormal corpus callosum morphology (14a).

### Comparison of males versus females and GOF versus LOF.

When comparing males and females with GOF variants, there was no difference in the frequency of epilepsy (7/7 males vs. 10/13 females, OR = ND; Fisher’s exact test, *P* = 0.52) (Figure 3B). However, males had an earlier median age of seizure onset at 4 months versus 36 months for females (Mann-Whitney test; *P* = 0.020). Given that some females did not develop seizures and males had earlier onset, the seizure risk profiles differed between sexes (Mantel-Cox test, χ^2^ = 9.9; *P* = 0.0016). The predominant seizure types were epileptic spasms in males and bilateral tonic-clonic seizures in females. Interestingly, there was no difference between the sexes regarding the likelihood of achieving seizure freedom (1/7 vs. 5/13, OR = 0.27 [95% CI: 0.020–2.4]; *P* = 0.35). However, males were more likely to have a broader range of comorbidities, including severe ID (5/7 vs. 3/13, OR = 8.3 [1.2–52]; *P* = 0.062), language problems (7/7 vs. 4/13, OR = ND; *P* = 0.0047), gait problems (6/7 vs. 1/12, OR = 66 [3.8–790]; *P* = 0.0017), scoliosis (5/7 vs. 2/13, OR = 14 [1.6–92]; *P* = 0.022), hypotonia (4/7 vs. 1/13, OR = 16 [1.3–210]; *P* = 0.031), CVI (4/7 vs. 0/13, OR = ND; *P* = 0.0072), and feeding problems (4/7 vs. 0/13, OR = ND; *P* = 0.0072). Finally, males were more likely to have inherited variants, while females were more likely to carry a de novo variant (6/7 vs. 1/11, OR = 54 [2.9–660]; *P* = 0.0037).

While all females with LOF variants were unaffected carriers, males had mild-to-moderate ID, language impairment, gait, and psychiatric problems. Epilepsy was observed in only 1 of 6 males. Thus, the clinical phenotypes of individuals with LOF variants are less severe than those with GOF variants, whether considering all individuals or comparing males and females separately.

**Figure 4:**
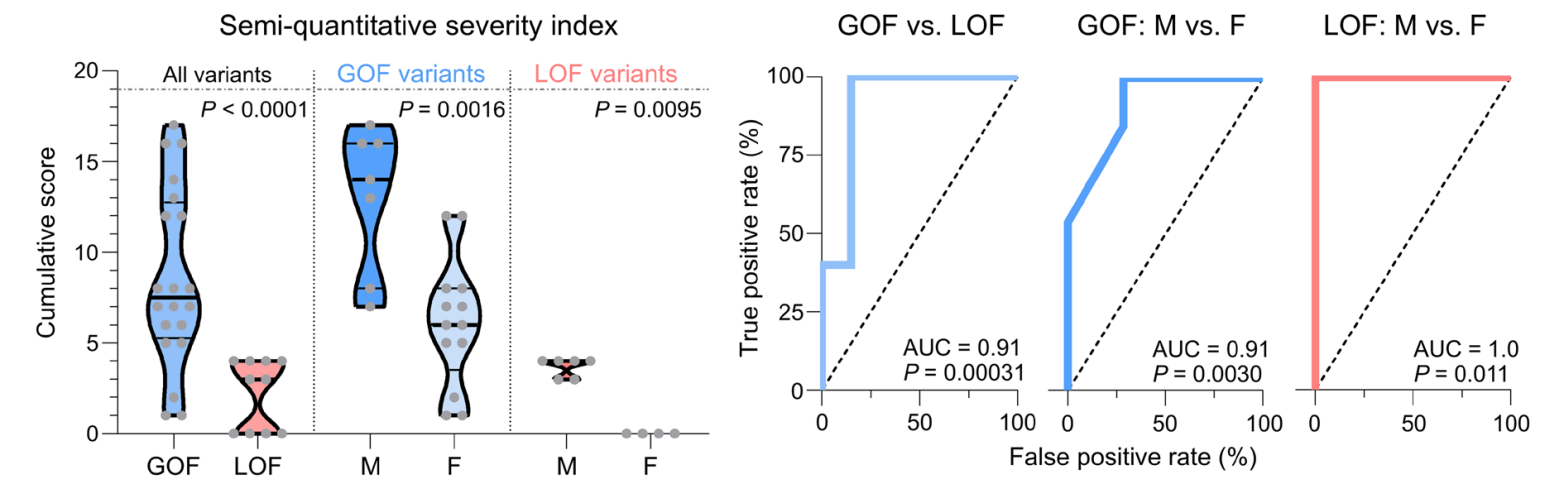
Semiquantitative phenotypic severity index. To model the overall phenotypic severity in each individual, cumulative scores were calculated for 30 individuals with GOF and LOF variants. Scores were derived by assigning values to 14 phenotypic features (as shown in Figure 3B and detailed in Supplemental Table 1). The maximum possible score is 19, indicated by the gray reference line. Statistical comparisons between subgroups were performed using the Mann-Whitney test, with corresponding *P* values shown. Receiver operating characteristic curves were generated to evaluate the performance of the severity index in distinguishing between GOF and LOF individuals, as well as between males and females within each variant category. The curves plot true positive rates against false positive rates across varying severity thresholds. The AUC is reported for each comparison, and statistical significance was assessed using the Wilson-Brown test, with *P* values indicated.

To further model these differences, a semiquantitative severity scale was employed to score the incidences across 14 phenotypic categories described above (see Methods and Supplemental Table 1 for details). The maximum possible score with this system was 19. When assessing all variants, the GOF group had a higher median score of 7.5 (IQR: 5.3–13) compared with 3.0 (IQR: 0–4) for the LOF group (Mann-Whitney test, *P* < 0.0001) (Figure 4). Receiver operating characteristic analysis indicated that the severity index was a strong predictor of GOF versus LOF molecular phenotype, with an AUC of 0.91 [95% CI: 0.80–1.0] (Wilson-Brown test, *P* = 0.00031). For GOF variants specifically, males had a median score of 14 [IQR: 8–16] compared with 6.0 [IQR: 3.5–8.0] for females (*P* = 0.0016). For LOF variants, males had a median score of 4 [IQR: 3–4] compared with 0 [IQR: 0–0] for females (*P* = 0.0095). Thus, the cumulative severity index is an effective method to model and differentiate the disease burden of the 4 groups.

### Treatment response analysis reveals limited therapeutic efficacy.

Apart from a single individual with a LOF variant, who was seizure free on levetiracetam (18a), antiseizure medication (ASM) was only used in the GOF group. Here, 3 individuals were seizure free: 2 females and 1 male. One male (5; mosaic for the GOF variant) achieved seizure freedom with levetiracetam, 1 female responded well to lamotrigine (12b), and another female was seizure free, although her ASM history was unclear (clobazam was reported; 2d). All other individuals had ongoing seizures despite trials of multiple ASMs. Several treatments, including lamotrigine, carbamazepine, valproate, gabapentin, lacosamide, and phenobarbital, were associated with either seizure worsening or severe side effects. Two individuals attempted the ketogenic diet: individual 5 experienced initial benefit but was unable to maintain it, while the other (12a) saw no improvement.

Cannabidiol was trialed in 2 individuals (9a and 9b) with minimal effect; however, both showed a remarkable response to cenobamate, achieving remission of tonic-clonic seizures and a substantial reduction in myoclonic seizures over the past 2 years. These findings underscore the urgent need for effective treatment strategies for individuals with *GABRA3*-associated disorders. The predominance of GOF variants suggests that targeted therapeutic approaches may be feasible. In other channelopathies, such as *SCN1A*-related disorders, treatment paradigms vary significantly depending on whether a variant leads to LOF or GOF (16). This precedent raises the possibility that sodium channel blockers, or other mechanism-specific interventions, could offer clinical benefit to some individuals with *GABRA3* variants.

### Gabra3 p.(Gln242Leu) mice are prone to seizures and premature death.

**Figure 5:**
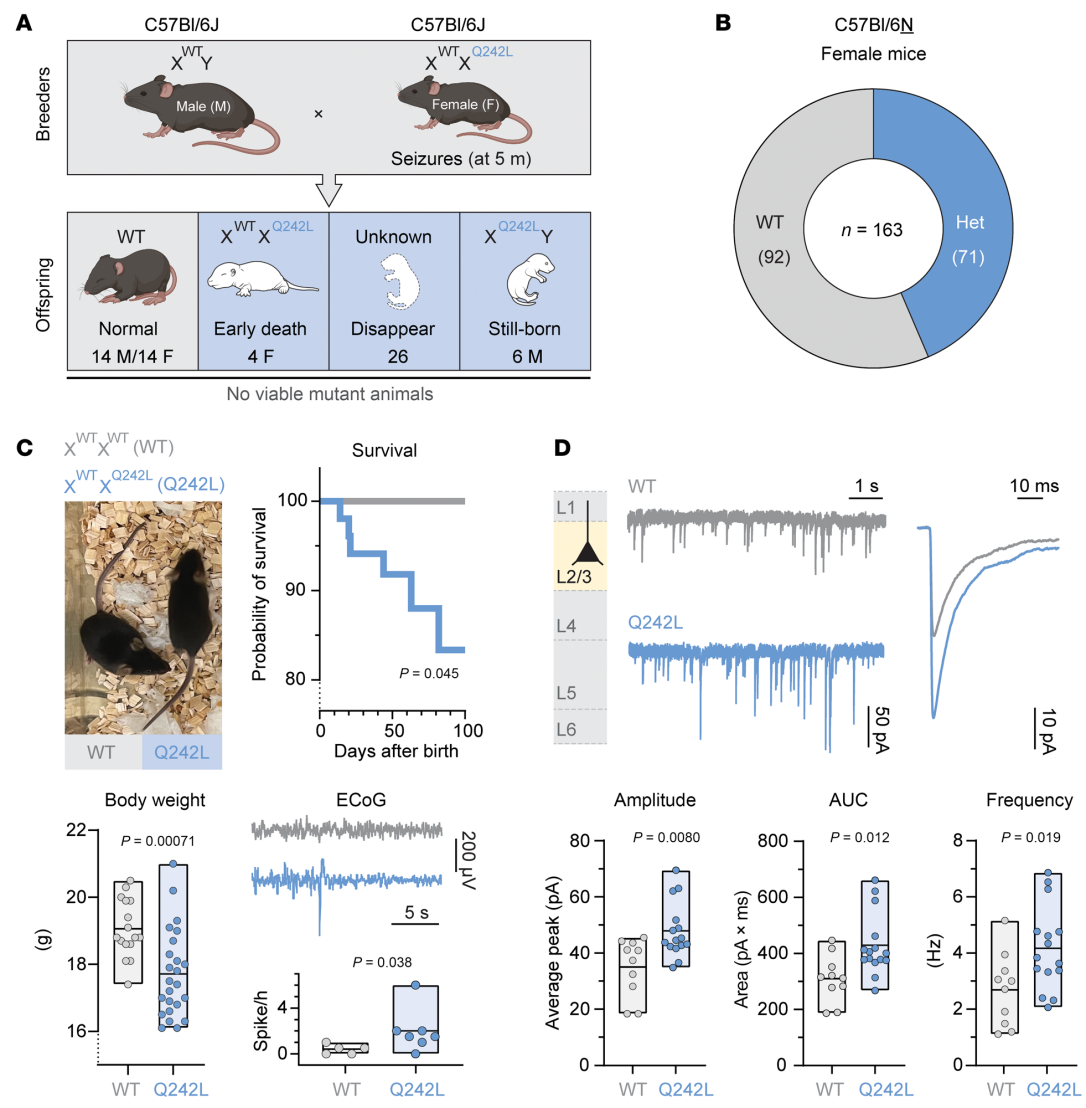
Phenotypic characterization of α3^Q242L^ mice. The *Gabra3* p.(Gln242Leu) knockin mouse model α3^Q242L/+^ (α3^Q242L^) was generated using CRISPR genome editing. (**A**) Genotype distribution of WT and α3^Q242L^ mice in the C57Bl/6J background. From a total of *n* = 64 mice (estimated based on early disappearance prior to genotyping), no live male α3^Q242L^ mice survived, while *n* = 4 live female mice heterozygous for the variant were obtained. (**B**) Genotype distribution is shown for a total of *n* = 163 female mice in the C57Bl/6N background. All subsequent analyses were conducted in female cohorts from the C57Bl/6N background. (**C**) Physical appearance and survival analysis are presented for α3^Q242L^ mice and WT littermates. Statistical significance was determined by a Mantel-Cox test. Body weight of *n* = 24 α3^Q242L^ mice compared with *n* = 16 WT littermates at 7 weeks of age is presented by individual measurements. Floating bars indicate the minimum and maximum obtained measurements, and the line signifies the mean weight. Statistical analysis was performed using a Mann-Whitney test. Representative ECoG traces are presented for α3^Q242L^ mice and WT littermates. Epileptiform spikes are presented as individual measurements for *n* = 7 α3^Q242L^ mice and *n* = 7 WT littermates. Statistical comparison was performed using a Mann-Whitney test. (**D**) mIPSCs were recorded from layer 2/3 cortical pyramidal neurons. Representative traces and overlaid averaged mIPSCs are shown for both genotypes. Peak current amplitudes, AUC, and frequency of spontaneous mIPSCs for *n* = 15 α3^Q242L^ mice and *n* = 10 WT are presented as individual measurements, with floating bars indicating the minimum and maximum obtained measurements and the line signifying the mean value. Statistical significance was determined using a Mann-Whitney test.

To model GOF *GABRA3* disease, a C57Bl/6J mouse with the p.(Gln242Leu) variant was engineered. After 4 rounds of zygote injections, 1 male mosaic founder and 7 female founders were obtained. From more than 60 next-generation (GenA) pups, only 5 live heterozygous females were obtained, with no surviving hemizygous males. The GenA female mice exhibited spontaneous seizures starting at 5 months (Supplemental Video 1), which was exacerbated by handling. Of an estimated 64 pups, no viable hemizygous males were produced, and only a few heterozygous females survived beyond P7. Many pups disappeared between birth and P7, likely due to maternal cannibalization of sick offspring (Figure 5A). Rare hemizygous males were stillborn, mirroring the more severe phenotype observed in human males.

To improve viability, the background strain was switched to C57Bl/6N. Even on this background, heterozygous *Gabra3*^Q242L/+^ mice (α3^Q242L^) were not born at Mendelian ratios. At P7, all male pups were WT, while 44% of female pups carried the variant (Figure 5B). Consequently, female mice were used for all subsequent experiments.

Surviving female α3^Q242L^ mice were smaller than WT littermates at week 7 (median 17.5 g [IQR: 16.7–18.8] vs. 18.8 g [18.6–19.9]; Mann-Whitney test, *P* = 0.00071) and exhibited higher mortality rate (Mantel-Cox test, χ^2^ = 4.0; *P* = 0.045) (Figure 5C). No substantial differences were observed in total brain weight or locomotor activity (Supplemental Figure 1). To assess brain hyperexcitability, electrocorticography (ECoG) recordings were performed in α3^Q242L^ mice aged 6–10 weeks. These mice showed increased epileptiform activity, with a higher frequency of spikes (Mann-Whitney test, *P* = 0.038), although ECoG power across the frequency spectrum remained unchanged under both light and dark conditions (Supplemental Figure 2).

Additionally, α3^Q242L^ mice displayed reduced latency to hind limb extension following pentylenetetrazole administration, indicating increased seizure susceptibility (Supplemental Figure 3).

Whole-cell electrophysiological recordings were performed on cortical layer 2/3 pyramidal neurons from α3^Q242L^ and WT mice (Figure 5D and Supplemental Figure 4). Miniature inhibitory postsynaptic currents (mIPSCs) were recorded at –70 mV in the presence of blockers of excitatory synaptic transmission and sodium channels (see Methods). Consistent with a GOF phenotype, α3^Q242L^ neurons showed a 37% increase in mIPSC amplitude (Mann-Whitney test, *P* = 0.0080), resulting in a 38% increase in charge transfer (*P* = 0.012). Additionally, mIPSC frequency was elevated by 55% (*P* = 0.019). Kinetic analysis revealed faster rise times, broader half-widths, and slower decay times, all consistent with GOF traits (Supplemental Figure 4).

## Discussion

This study adds *GABRA3* to a growing list of GABA_A_ receptor subunit genes for which both GOF and LOF missense variants have been observed. This list currently includes *GABRA1* (6, 17), *GABRA4* (18), *GABRB1-3* (7–10), *GABRD* (11), and *GABRG2* (12) and is likely to expand with additional subunits in the future. Consistent with recent findings, distinct differences in clinical features were noted between GOF and LOF *GABRA3* groups. However, due to its location on the X chromosome, pathogenic variation in *GABRA3* is unique, causing markedly divergent disease patterns between the sexes.

While GOF variants were observed in both hemizygous males and heterozygous females, males generally had more severe phenotypes. Among the 7 males, all had DEE with a median seizure onset age of 4 months (Figure 3B). Notable features included language, gait, and cortical visual impairments, scoliosis, and hypotonia. Most males had severe ID, with cognitive impairment often present before seizure onset. By contrast, 10 of the 13 females experienced seizures with a later median onset age of 3 years. While only a few had severe ID or many of the features observed in males, other neuropsychiatric features such as anxiety or autistic behavior were frequently observed. This sex-dependent difference in disease severity and manifestations can be explained by the difference in X chromosome copies, which affects α3-containing receptor compositions. Consistent with previous studies, additive effects were noted with the introduction of an additional variant subunit, with double-variant receptors typically exhibiting twice the change in GABA sensitivity (19, 20). Therefore, the functional impact of a GOF variant is markedly more severe in males, likely explaining the increased severity of disease.

An engineered mouse model carrying the GOF p.(Gln242Leu) variant recapitulated key human findings (Figure 5). Breeding of these mice was challenging, and despite various strategies, no live hemizygous males were obtained at P7, indicating that males are considerably more severely affected. Whilst it cannot be excluded that live hemizygous male pups were born, they likely exhibited abnormal behavior, prompting the dam to cannibalize them. Given that 4/7 human male infants had feeding difficulties, it is possible that any live-born hemizygous pups were unable to initiate feeding. Female mice showed increased mortality in the first 2 months of life and developed spontaneous seizures in adulthood. Consistent with a GOF phenotype, electrophysiological analysis of cortical pyramidal neurons revealed increased current amplitudes of spontaneous mIPSCs. This GOF mouse is thus a more representative model of the severe form of the disease than *Gabra3*-KO mice (with LOF), in which early mortality or seizures are not reported (5).

A remarkable difference between the GOF and LOF human patients lies in the occurrence of epilepsy, with individuals with LOF variants rarely having seizures (Figure 3). Only 1 male proband, who was very young at the time of data collection, had epilepsy and became seizure free. The remaining 5 male probands and 4 mothers carrying LOF variants did not have seizures. Despite the relatively small number of individuals in the LOF group, this observation gains strength from its basis in both protein-truncating variants and de novo missense variants with functional LOF traits. Nonetheless, the male probands with LOF variants had a range of other conditions such as language delay, dysmorphic features, and behavioral disorders. Some of these features were also seen in the GOF group but were less severe in the LOF cohort. For example, in the LOF group, no individuals were nonverbal, although delayed language development was observed. Similarly, 5 individuals with LOF variants had mild-to-moderate ID (and 1 had normal intellect) as opposed to mainly severe ID in the males with GOF variants. Finally, it is noteworthy that all affected individuals with LOF were males, and in those where the variant was inherited from a mother, the mother was unaffected.

In X-linked disorders, affected males can inherit a pathogenic allele from an unaffected female carrier in recessive X-linked disorders, while both males and females are affected in dominant X-linked disorders. This interpretation can, however, be confounded by X inactivation patterns, where WT allele expression is suppressed in females, leading to variable penetrance of the phenotype. For the pathogenic *GABRA3* variants, a distinct and consistent sex-linked association of neurodevelopmental disorders is observed, which correlates with the functional analysis findings and is not suggestive of random X inactivation in females. In GOF *GABRA3* variants, affected individuals present with moderate-to-severe epilepsy in all hemizygous males and most heterozygous female carriers. Although the overall phenotype is milder, females are still affected (Figure 4). This suggests that GOF *GABRA3* variants behave as a dominant X-linked disorder whereby overactive α3-containing GABA_A_ receptors are penetrant also in most/all females. In stark contrast, neurodevelopmental disorders are present in hemizygous males with a LOF variant, but not in heterozygous female carriers. This suggests that LOF *GABRA3* variants behave as a recessive X-linked disorder and are nonpenetrant in females. These observations are critical in interpreting the phenotypes of *GABRA3* variations and make clinical counseling of the families more complex.

The core phenotype of *GABRA3*-related disorders in this study, relating to seizures and developmental impairment, correlated with the functional outcome of each specific variant. Similar observations have been reported for other GABA_A_ receptor genes, such as *GABRB2* (8) and *GABRB3* (9). In all cases, individuals with GOF variants presented with a more severe phenotype compared with individuals with LOF variants. While the phenotypic spectrum of *GABRA3* disease resembles that of other synaptic GABA_A_ receptors, it also shows notable differences. For example, females with heterozygous *GABRA3* GOF variants have a later age of seizure onset compared with those with *GABRB3* variants (median 36 vs. 2.5 months). Additionally, higher rates of visual impairment and nystagmus were seen in the *GABRA3* GOF group, likely related to the restricted distribution of the α3 subunit in the cerebral cortex, including the occipital cortex (2). Thus, while there are phenotypic similarities between individuals with GABA_A_ receptor variants, important differences arise from the distinct physiological roles of the receptor subtypes. Visual abnormalities were generally described as cortical visual impairment; however, it is noteworthy that α3 subunits are found in the eye, particularly in developing retinal bipolar cells during the formation of presynaptic inhibitory synapses (21). Hence, the origins of visual deficits should be further investigated.

Unlike other GABA_A_ receptor subunits, very few individuals with LOF variants were identified. This scarcity may result from underdiagnosis of inherited LOF variants, as genetic testing may not be routine for individuals with less severe ID only or because LOF variants are better tolerated for *GABRA3*. Other special aspects of *GABRA3* variants include their relatively low population frequency and the predominance of de novo variants in females. Even severe GOF variants in males are more likely to be inherited. These patterns are likely due to the location of *GABRA3* on the X chromosome and differing rates of paternally and maternally inherited de novo variants. In a study of over 15,000 de novo variants from 1,548 trios in Iceland, approximately 80% were of paternal origin (22). The rate of de novo variants increased with paternal age, presumably due to repeated mitosis of spermatogonia. Since females inherit both a paternal and maternal X chromosome, and males only inherit a maternal X chromosome, the likelihood of a *GABRA3* de novo variant being observed in an affected female is substantially higher. Consequently, the population frequency of *GABRA3* variants is lower because there are fewer opportunities for de novo variants to arise from the paternal line. Males, inheriting only 1 X chromosome, are more likely to present with inherited variants compared with other GABA_A_ receptor genes.

This study has several limitations. Variants in *GABRA3* are rare, limiting the number of identified affected individuals for study. This issue is exacerbated by the need to segregate data based on both functional outcomes and sex. Consequently, sparse-data bias may occur, which for rare comorbidities may lead to unrealistically large OR CIs or indeterminable estimates with complete penetrance in 1 group. Additionally, the study relied on retrospective data collected by clinicians from medical records, patient interviews, and the literature. This approach introduces heterogeneity in data collection and analysis, potentially affecting comprehensive phenotypic descriptions. The use of an in vitro overexpression system also carries inherent limitations. These models may not accurately reflect the complexity of neuronal environments or incorporate key modulatory influences on receptor function. In this study, the assay targeted 2 well-established functional parameters; however, other potentially important aspects, such as receptor trafficking and desensitization, were not evaluated in detail. Nonetheless, the observed increase in mIPSC amplitudes in *Gabra3*^Q242L/+^ mouse neurons provides compelling evidence that the in vitro expression system used here reliably predicts GOF phenotypes in situ.

### Conclusion.

Our findings demonstrate that diverging functional effects of genetic variants can influence whether an X-linked disorder manifests as dominant or recessive. This may have broader implications for other X-linked genes where pathogenic variants lead to divergent functional outcomes. Indeed, the data suggest that GOF molecular phenotypes may be more penetrant and severe in other genes associated with DEE. Although *GABRA3* is not yet routinely included in smaller epilepsy gene panels, the increasing adoption of exome and genome sequencing will enhance the detection of pathogenic *GABRA3* variants in the future. While LOF variants are not directly linked to epilepsy, they are associated with mild ID and behavioral disorders such as ADHD and ASD in males. Therefore, it is essential not to overlook inherited *GABRA3* variants during genetic workup. Additionally, sequencing unaffected mothers may yield valuable insights and support more informed genetic counseling.

## Methods

### Sex as a biological variable

Our study included male and female individuals, and sex-dimorphic effects are reported. Our study also examined male and female mice, and sex-dimorphic effects are reported.

### Patient cohort

Individuals with variants in *GABRA3* were recruited through an international collaboration with epilepsy and genetic centers, including EpiCARE, the European Reference Network for Rare and Complex Epilepsies. Additional individuals were recruited through GeneMatcher (23). Data were collected in a standardized manner in a REDCap database hosted at the Danish Epilepsy Centre (24, 25) and included information on the variant, family history, epilepsy phenotype, development, EEG reports, and treatment response. Probands and family members carrying the variant were included in the study. All variants were classified according to ACMG guidelines (26). Previously unpublished individuals with variants in *GABRA3* were included in the study regardless of previous classification. The transcript NM_000808.4 was used. Seizures were classified according to the most recent guidelines from the International League Against Epilepsy (27). For all clinical characteristics, human phenotype ontology terms were used whenever possible to make data comparable across individuals. ID was stratified into the following categories: normal, mild, moderate, and severe/profound. Sex was accounted for as a biological variable by separating into male and female groups for the LOF and GOF cohorts. Functional studies were performed to replicate the different receptor subtypes likely to exist in male and female patients.

### Cumulative severity index

To capture the overall impact across multiple phenotypic features, we developed a holistic disease burden measure. For each individual, a severity index was calculated as the sum of scores for all phenotypic features. The following scores were assigned to the various phenotypic features: inheritance (0 for inherited, 1 for de novo), epilepsy (0 for no, 1 for yes), age of seizure onset (0 for no epilepsy, 1 for onset above 1 year, 2 for onset less than 1 year), seizure frequency (0 for seizure free; 1 for monthly, yearly, or ongoing frequency unknown; 2 for daily or weekly), DD/ID (0 for normal, 1 for mild to moderate, 2 for severe to profound), language delay (0 for no delay, 1 for delay, 2 for nonverbal), gait (0 for normal, 1 for gait issues, 2 for nonambulant), nystagmus (0 for no, 1 for yes), CVI (0 for no, 1 for yes), scoliosis (0 for no, 1 for yes), hypotonia (0 for normal, 1 for hypotonia), ASD/ADHD/aggressive behavior (0 for no, 1 for yes), feeding difficulties (0 for no, 1 for yes), and microcephaly (0 for no, 1 for yes). Further details regarding scoring are presented in Supplemental Table 1.

### Functional studies

Pentameric α3β3γ2 GABA_A_ receptors consist of 2 α3, 2 β3, and 1 γ2 subunit(s). This cohort includes both heterozygous and hemizygous patients carrying respective variants. To assess the function of receptors that have 1 WT α3 and 1 variant α3 subunit as well as receptors that have 2 variant α3 subunits, subunit concatenation is the most reliable methodology. Therefore, pentameric concatenated GABA_A_ receptor constructs were created with either 1 or 2 mutated α3 subunits that are efficiently expressed in *Xenopus laevis* oocytes (13, 14). In total, 31 pentameric α3β3γ2 GABA_A_ receptor concatenated constructs were generated: 1 WT construct, 15 single-mutant constructs containing a mutated and a WT α3 subunit, and 15 double-mutant constructs containing 2 mutated α3 subunits (Figure 1A). For functional analysis, the cRNAs of concatenated receptors along with the WT (control) were injected into oocytes (25 ng/oocyte) and subsequently evaluated using two-electrode voltage clamp electrophysiology as previously described (13, 14). These experiments were conducted using a custom-built apparatus that records receptor responses to GABA. Interday variation between oocyte batches were controlled by performing WT experiments in parallel to variants on each experimental day. GABA concentration–response curves (*n* > 10) were obtained by applying increasing concentrations of GABA to each oocyte. Maximum current amplitudes (I_max_, *n* > 20) were determined by applications of 10 mM GABA solution and normalized against the WT value of the day. All experiments were conducted in at least 2 batches of oocytes from different animals.

To obtain the EC_50_ values (the concentration that elicits 50% of maximum response) from the GABA concentration–response curves, the Hill equation was fitted to the GABA-evoked current amplitudes for individual oocytes, where *n*H is the Hill slope:

 Equation 1


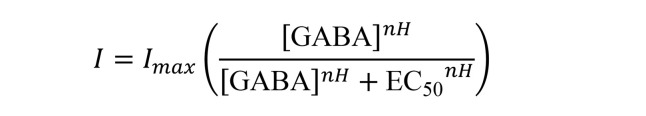


Responses were normalized to the fitted maximum response of individual curves. Full concentration–response curve of individual oocytes was recorded as a single determination (*n*). Average logEC_50_ values for the WT control [logEC_50_(WT)] were calculated, and the ΔlogEC_50_ values for each variant experimental determination on the same day were derived by the equation ΔlogEC_50_ = logEC_50_(WT) − logEC_50_.

For functional analysis, a ΔlogEC_50_ value of ≥ 0.2 typically indicates a GOF variant, whereas a value ≤ −0.2 indicates a LOF variant. The higher the absolute value, the greater the change in GABA sensitivity.

### Animal behavior studies

The *Gabra3* p.(Gln242Leu) knockin mouse model was produced via CRISPR genome editing by the Monash Genome Modification Platform, Monash University, as a node of Phenomics Australia. Phenomics Australia is supported by the Australian Government Department of Education through the National Collaborative Research Infrastructure Strategy, the Super Science Initiative, and the Collaborative Research Infrastructure Scheme.

#### Animal ethics, housing, and husbandry.

All experiments were performed in accordance with the Prevention of Cruelty to Animals Act, 1986, under the guidelines of the National Health and Medical Research Council of the Australian Code of Practice for the Care and Use of Animals for Experimental Purposes. Mice were generated at Monash Genome Institute on a C57Bl/6J background. After several failed litters, a few surviving mice were transferred to the mouse facility at the Florey Institute of Neuroscience and Mental Health. Due to the extreme difficulty in breeding, the mice were then bred on C57Bl/6N background and all the experiments were done on this background and only on females due to unavailability of male knockin mice. The mice were group-housed in standard 15 × 30 × 12 cm cages under a 12-hour dark/light cycle and had access to dry pellet food and tap water ad libitum.

All experiments were approved by the Animal Ethics Committee at the Florey Institute of Neuroscience and Mental Health. Animals were monitored in line with protocols approved by the committee. Anesthesia and analgesia were used where appropriate. Mice were acclimatized to experimental rooms for at least 1 hour before experimentation. At the conclusion of experimentation, mice were culled by cervical dislocation or decapitation following deep anesthesia, which are methods approved by the Australian & New Zealand Council for the Care of Animals in Research and Teaching.

#### ECoG electrode implantation surgery and recordings.

Female WT (*n* = 7) and heterozygous littermate α3^Q242L^ mice (*n* = 7) were used for ECoG experiments to study baseline epileptiform activity. ECoG electrode implantation was performed at >P40 as previously described (also see Supplemental Methods) (28). Briefly, mice were anesthetized, and the head was secured under the stereotaxic frame. Under microscope, the reference, ground, and 2 active channel electrodes were secured to the skull and then soldered to a mouse electroencephalography head mount (8201-EEG, Pinnacle Technology). Self-curing acrylic resin was used to hold the head mount and electrodes in place. Mice were allowed to recover for at least 1 week prior to experimentation. ECoG data were sampled at 250 Hz and filtered (40 Hz low pass, 0.5 Hz high pass) using Sirenia Acquisition software (version 2.1.0, Pinnacle Technology). Recordings were conducted for 24 hours to characterize baseline ECoG activity.

#### Spike counting.

Spike analysis was performed by visual inspection of raw ECoG data extracted using Sirenia Seizure Pro software (version 1.7.5, Pinnacle Technology). Spikes had distinctive morphology and were defined as biphasic events lasting < 200 ms with at least twice the amplitude of baseline ECoG activity. Spikes in 2 hours were counted to study any underlying epileptiform activity. All analyses were completed by an experimenter blinded to the categories of mice.

#### Behavioral assays.

Mice were allowed to acclimatize in their home cages to the behavioral room for at least 1 hour prior to the commencement of any behavioral assay. Mice were assessed using the open-field locomotor test.

#### Brain slice electrophysiology.

Acute brain slice electrophysiology was performed from P21–P34 mice as previously described (28). Briefly, mice were anesthetized and decapitated, and the extracted brain was prepared into 300 μm coronal slices containing the cortex using a vibratome (Leica VT1200S). The brain slices were kept in a holding chamber at 32°C for 45 minutes and maintained at room temperature. The slices were then transferred to the recording chamber and viewed under ×40 water immersion objective (Olympus). Whole-cell patch clamp recordings were made from layer 2/3 cortical pyramidal neurons in voltage clamp mode (–70 mV holding potential) to measure the spontaneous mIPSCs. The glass pipettes (6–8 MΩ) were filled with the internal solution containing (in mM) 135 KCl, 7 NaCl, 10 HEPES, 0.1 EGTA, 2 MgCl_2_, 2 Na_2_·ATP, 0.3 Na_3_·GTP, 5 QX314-Cl, and 0.2% biocytin (290–300 mOs/kg, pH 7.2). The external artificial cerebrospinal fluid (aCSF) (32°C–34°C, maintained at ~3–4 mL/min) was supplemented with 10 μM cyanquixaline, 20 μM DL-AP5, and 0.5 μM tetrodotoxin to block AMPA and NMDA receptors as well as voltage-gated sodium channels, respectively. In some experiments, aCSF containing 100 μM Picrotoxin was used to confirm the isolation of mIPSCs. Series resistance compensation was performed at 60%–75%.

The recordings were made using an Axon Multiclamp 700B amplifier, Digidata 1440A digitizer (Molecular Devices), and pCLAMP version 10 software (Molecular Devices). The data were sampled at 50 kHz with a low-pass filter at 10 kHz and analyzed using Axograph X (version 1.8.0; Axograph Scientific). The mIPSCs were detected by a template created using a SD of 4.5–5. The peak mIPSC amplitude was measured from the baseline. Halfwidth was defined as the width of the mIPSCs halfway between the baseline and the peak. Rise time was measured from 10% to 90% of the mIPSC amplitude. Decay time was measured from 100% to 50% of the mIPSC amplitude. The area (charge transfer) was calculated from the AUC of the isolated mIPSCs. The average frequency was calculated as mIPSC events per second.

#### Exclusion criteria.

For slice electrophysiology data, cells were excluded from analysis if access resistance exceeded >30 MΩ at any time point or if it fluctuated by more than 20% during the recording session. For mouse behavioral and EEG experiments, data > 2 SD away from the mean were excluded.

### Statistics

Age of seizure onset was compared using both the Mann-Whitney *U* test and the Mantel-Cox test to assess seizure risk over time. Categorical data for individuals with LOF and GOF variants, as well as sex differences within each group, were analyzed using 2-sided Fisher’s exact tests. A significance threshold of *P* < 0.05 was applied. CIs for ORs were calculated using the Baptista–Pike method. GABA concentration–response curves were generated by fitting individual experimental data to the Hill equation and analyzed using nonlinear regression in GraphPad Prism 10. Differences in GABA sensitivity (ΔlogEC_50_ values) were assessed using 1-way ANOVA with Dunnett’s post hoc test corrected for multiple comparisons. I_max_ values were compared using the Mann-Whitney *U* test. For both ΔlogEC_50_ and I_max_ values, a conservative significance threshold of *P* < 0.0001 was used. All statistical analyses for in vivo mouse data were conducted using GraphPad Prism 10. Comparisons between α3^Q242L^ mice and WT littermates were performed using the Mann-Whitney *U* test. Survival analyses were conducted using the Mantel-Cox test.

### Study approval

This study was approved by the IRB of the Danish Epilepsy Centre, Filadelfia (Project ID EMN-2024-01998).

### Data availability

All available data are presented in the main manuscript, the supplemental files, or the Supporting Data Values file.

## Author contributions

Conceptualization: KMJ, MC, RSM, CAR, and PKA. Methodology: KMJ, KPA, VWYL, NA, HCC, RSM, CAR, and PKA. Investigation: KMJ, KPA, VWYL, NA, HCC, XNG, MM, CEM, HML, SO, RCS, VS, RAR, GMM, PAW, JFD, CH, PBT, DD, JZ, NB, YC, BK, MS, IMBHVDL, MAVS, RP, MB, DKB, EA, LC, NQ, HE, IMW, MCK, PB, CIGM, VR, DB, KA, JCW, CEJ, PZ, KP, SFB, IES, MC, GR, RSM, CAR, and PKA. Funding acquisition, project administration, and supervision: MC, RSM, CAR, and PKA. Writing (original draft): KMJ, KPA, NA, and PKA. All authors have reviewed the final manuscript.

## Funding support

This work is the result of NIH funding, in whole or in part, and is subject to the NIH Public Access Policy. Through acceptance of this federal funding, the NIH has been given a right to make the work publicly available in PubMed Central.

- The Australian National Health and Medical Research Council (grant APP2019780 to PKA, MC, CAR, VWYL, and RSM).
- The Lundbeck Foundation (grant R383-2022-276 to RSM and PKA).
- The National Institute of Neurological Disorders and Stroke of the NIH (award U01HG007672 to RCS).
- Stiftung Michael funding through the assistance of the Canger-Janz-Fellowship (to PZ).
- DFG funding (project 499552394, SFB 1597; project 503306912, FOR 5547; project 431984000, SFB 1453; to MS).
- The Cluster of Excellence, CIBBS – Centre for Integrative Biological Signalling Studies (EXC-2189, project ID 390939984; to MS).

## Supplementary Material

Supplemental data

Supplemental table 1

Supplemental video 1

Supporting data values

## Figures and Tables

**Table 1:**
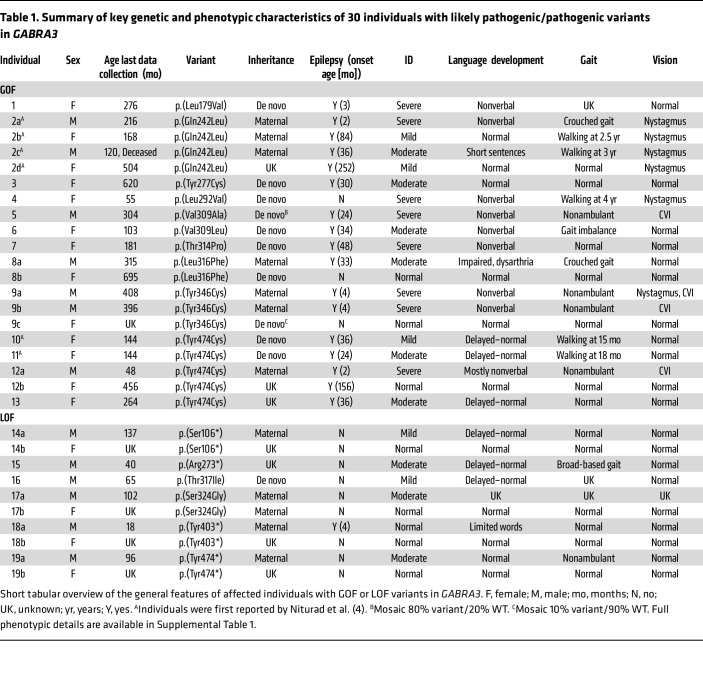
Summary of key genetic and phenotypic characteristics of 30 individuals with likely pathogenic/pathogenic variants in *GABRA3*

## Version 1 11/25/2025

In-Press Preview

## Version 2 01/16/2026

Electronic publication
