# Supplementary material for: Functional consequence of pathogenic GABRA3 variants determines whether X-linked inheritance is dominant or recessive
Source: J Clin Invest. 2025 Nov 25;136(2):e189830. doi: 10.1172/JCI189830 (PMC12807467; doi:10.1172/JCI189830)
Supplement: Supplemental data [file jci-136-189830-s245.pdf]

Functional consequence of pathogenic *GABRA3* variants determines whether X-linked inheritance is dominant or recessive

Supplementary information

Katrine M. Johannesen†, Khaing Phyu Aung†, Vivian W. Y. Liao†, Nathan L. Absalom, Han Chow Chua, Xue Ning Gan, Miaomiao Mao, Chaseley E. McKenzie, Hian Mun Lee, Mary Chebib, Guido Rubboli, Rikke S. Møller*, Christopher A. Reid*, and Philip K. Ahring*.

†These authors contributed equally to this work.

*Corresponding authors.

Contents

Supplementary Figure 1. Brain weight and locomotion of $\alpha3^{Q242L}$ mice and WT littermates. 2

Supplementary Figure 2. ECoG power spectra of $\alpha3^{Q242L}$ mice and WT littermates. 3

Supplementary Figure 3. Seizure susceptibility of $\alpha3^{Q242L}$ mice and WT littermates. 4

Supplementary Figure 4. $GABA_A$-mediated synaptic currents in cortical layer 2/3 pyramidal neurons of $\alpha3^{Q242L}$ mice and WT littermates. 5

Supplementary Methods. Detailed methods for electrocorticography electrode implantation surgery and recordings 6

Supplementary Figure 1. Brain weight and locomotion of α3^{Q242L} mice and WT littermates.

To assess whether the α3^{Q242L} mice exhibit differences in brain weight compared to their wildtype (WT) littermates, brains were extracted and weighed from 7 WT and 7 α3^{Q242L} mice. No differences in brain weight were observed between the two genotypes (Supplementary Figure 1A).

To evaluate potential differences in behavioural patterns, a locomotion test was conducted. The test revealed no noticeable differences between genotypes in total distance travelled, total jump count, or total movement time over a 60-minute period, indicating comparable locomotor activity (Supplementary Figure 1B, 1C).

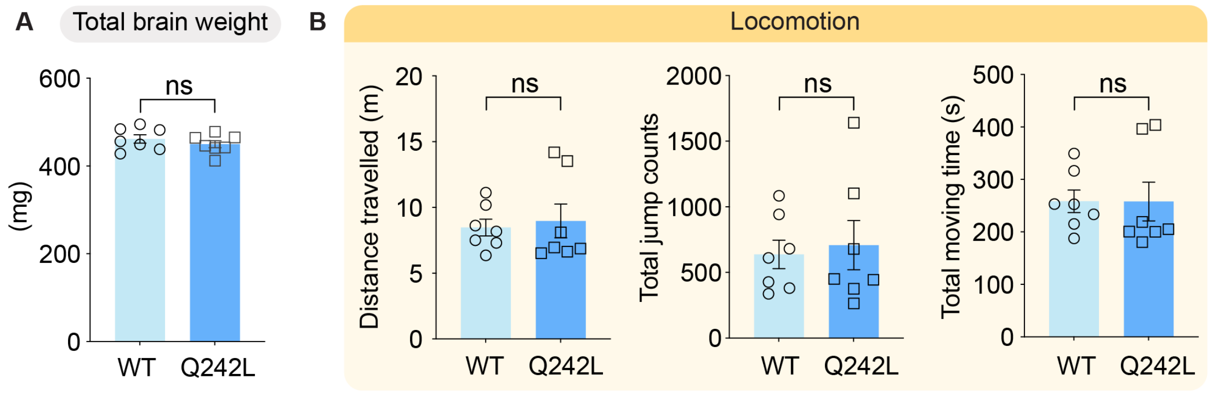

C

Parameter	WT (n=7)	Q242L (n=7)	*P*-value	Statistic used
Distance travelled (m)	8.47 ± 0.63	8.98 ± 1.28	0.71	Mann Whitney test
Total jump counts	638 ± 109	708 ± 187	1	Mann Whitney test
Total moving time (s)	258 ± 57	258 ± 98	0.54	Mann Whitney test
Resting time (s)	2460 ± 50	2250 ± 200	0.90	Mann Whitney test
Average speed (mm/s)	38.9 ± 1.5	43.2 ± 1.8	0.073	Mann Whitney test

Supplementary Figure 1. Brain weight and locomotion test of WT and α3^{Q242L} mice. (A) comparison of total brain weight between WT and α3^{Q242L} littermates, P = 0.36, unpaired *t*-test. **(B)** Locomotor test results showing comparisons of total distance travelled, total jump counts, and total moving time over a 60-minute period. Bars represent mean observations with ± SEM along with individual observations for n = 7 WT and n = 7 α3^{Q242L} mice. **(C)** Detailed information for key locomotor parameters and statistical analyses.

Methods. All experiments are from female mice using the C57Bl/6N background. Mice (P40 – P60) were placed in a square 27.3 ×27.3 × 20.3 cm open-field arena and were allowed to move freely for one hour, during which infrared rays tracked their movement. Data indicating distance travelled and other parameters was recorded and compiled using MED Associates Activity Monitor software (Med Associates Inc.).

Supplementary Figure 2. ECoG power spectra of α3^{Q242L} mice and WT littermates.

To determine whether α3^{Q242L} mice and WT littermates exhibit differences in ECoG power spectra, 24-hour EEG recordings were conducted, and spectral analyses were performed during both light and dark phases. No differences were observed in ECoG spectral power across all four power bands (delta, theta, alpha, and beta) in either phase (Supplementary Figure 2).

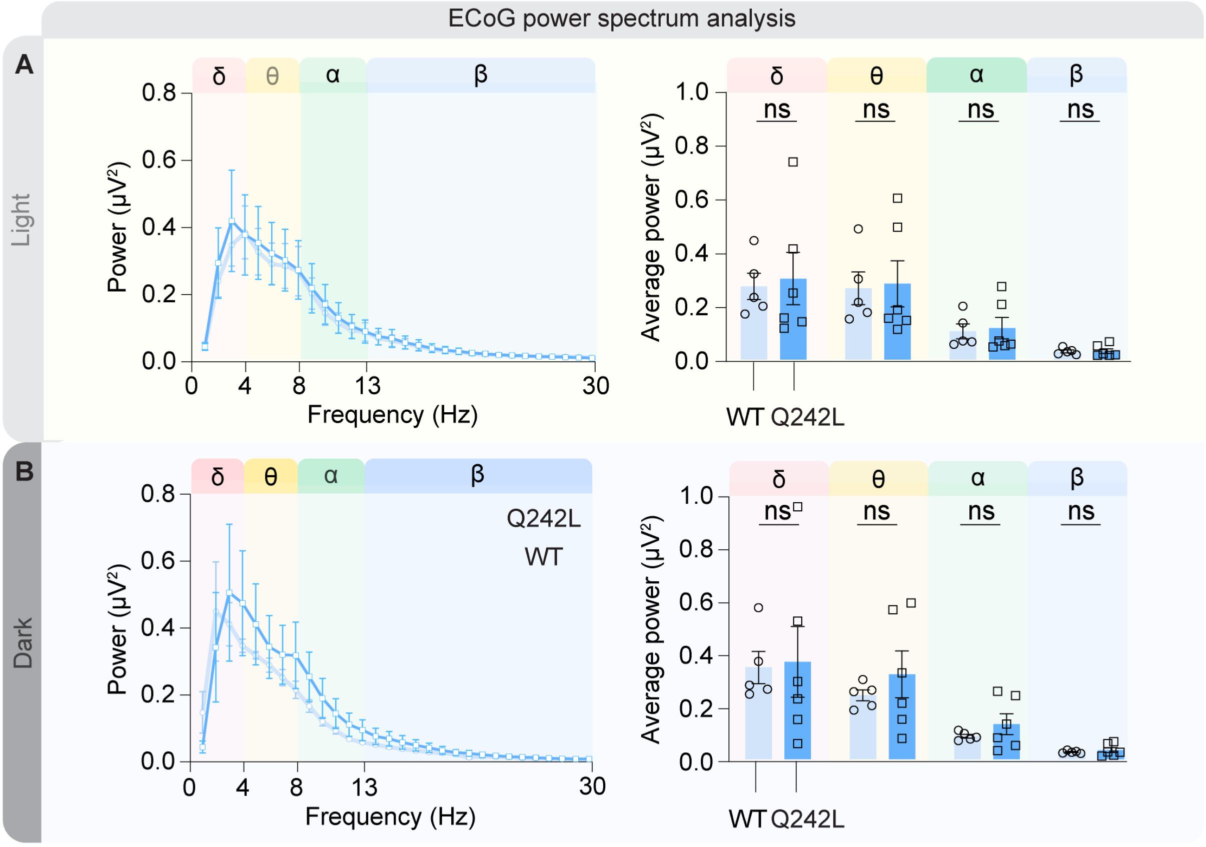

Supplementary Figure 2. ECoG power spectra of WT and α3^{Q242L} mice. (A) ECoG spectral power analysis across all four power bands in the light phase: delta $P > 0.9999$, theta $P = 0.84$, alpha $P > 0.9999$ and beta $P > 0.9999$, Mann Whitney test. **(B)** ECoG spectral power comparison in the dark phase: delta $P = 0.55$, theta $P > 0.9999$, alpha $P > 0.9999$ and beta $P > 0.9999$, Mann Whitney test. Data are presented as mean ± SEM for n = 5 WT and n = 6 α3^{Q242L} mice. All experiments are from female mice using the C57Bl/6N background.

Supplementary Figure 3. Seizure susceptibility of α3^{Q242L} mice and WT littermates.

To determine whether α3^{Q242L} mice exhibit increased seizure susceptibility relative to WT littermates, we employed a standard proconvulsant assay using the $GABA_A$ receptor antagonist pentylenetetrazole (PTZ). Following subcutaneous injection of PTZ (100 mg/kg), the latency to two well-defined seizure endpoints were recorded: the onset of the first tonic-clonic seizure and hindlimb extension. While the latency to the first tonic-clonic seizure did not differ between groups (Supplementary Figure 3A), the α3^{Q242L} mice exhibited a shorter latency to hindlimb extension (Supplementary Figure 3B), indicating heightened seizure susceptibility compared to their WT littermates.

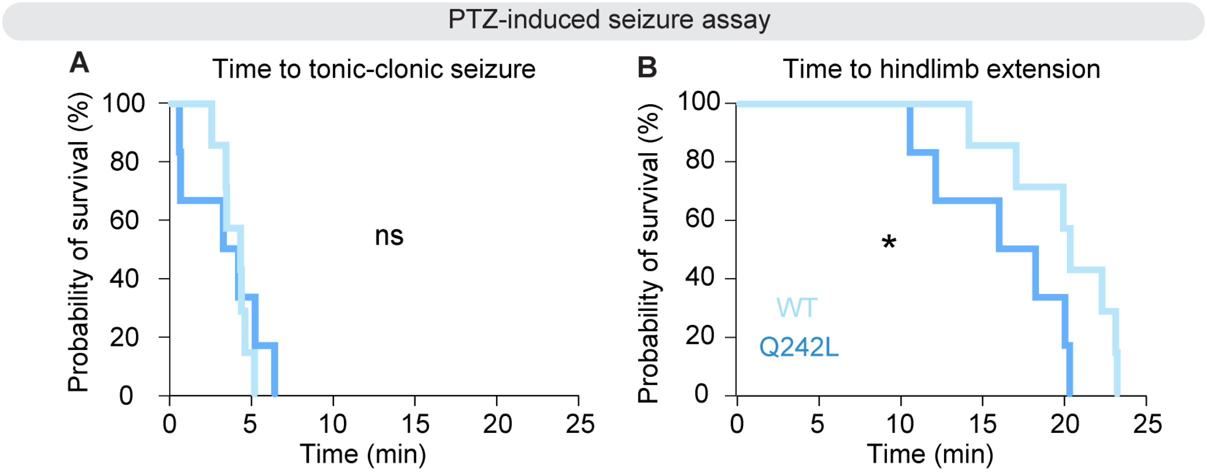

Supplementary Figure 3. PTZ-induced seizure testing in WT and α3^{Q242L} mice. (A) Time to first tonic-clonic seizure compared between WT and α3^{Q242L} littermates after the subcutaneous PTZ injection, P = 0.67, Mantel-Cox Log-Rank test. **(B)** Time to hindlimb extension in the same mice, *P = 0.046, Mantel-Cox Log-Rank test, n = 7 WT and n = 6 α3^{Q242L} mice. All experiments are from female mice using the C57Bl/6N background.

Supplementary Figure 4. $GABA_A$-mediated synaptic currents in cortical layer 2/3 pyramidal neurons of $\alpha3^{Q242L}$ mice and WT littermates.

To assess whether the altered biophysical properties of $\alpha3^{Q242L}$-containing $GABA_A$ receptors manifest at the synaptic level, we conducted whole-cell patch-clamp recordings from layer 2/3 pyramidal neurons in the cortex of $\alpha3^{Q242L}$ and WT mice. Miniature inhibitory postsynaptic currents (mIPSCs) were recorded at a holding potential of –70 mV. Compared to WT, $\alpha3^{Q242L}$ neurons exhibited 15% faster rise times (Supplementary Figure 4A), 17% broader halfwidths (Supplementary Figure 4B), and 50% slower decay times (Supplementary Figure 4C). These findings align with the GOF properties observed in $\alpha3^{Q242L}$ mutant receptors expressed in oocytes, suggesting that these synaptic alterations reflect the underlying receptor-level changes.

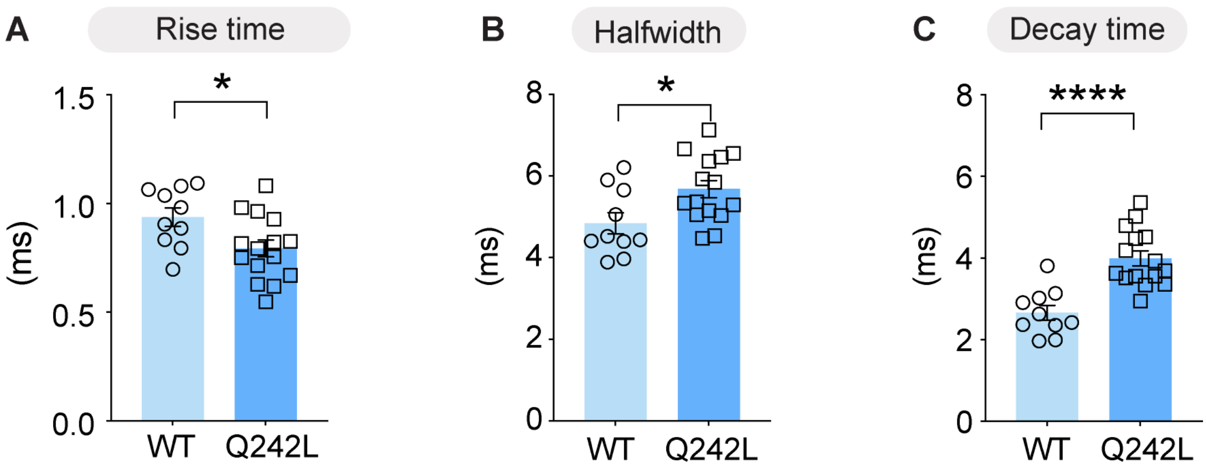

D

Parameter	WT (n=10)	Q242L (n=15)	*P*-value	Statistic used
Peak amplitude (pA)	35.0 ± 3.3	47.9 ± 2.6	0.0080	Mann Whitney test
Area (pA*ms)	309 ± 27	428 ± 29	0.012	Mann Whitney test
Frequency (Hz)	2.69 ± 0.41	4.17 ± 0.39	0.019	Mann Whitney test
Rise time (ms)	0.937 ± 0.043	0.793 ± 0.038	0.022	Unpaired t-test
Half width (ms)	4.85 ± 0.26	5.68 ± 0.21	0.019	Unpaired t-test
Decay time (ms)	2.66 ± 0.18	3.99 ± 0.18	<0.0001	Unpaired t-test

Supplementary Figure 4. Comparison of mIPSCs characteristics between WT and $\alpha3^{Q242L}$ mice. (A) Averaged mIPSCs rise time compared between WT and $\alpha3^{Q242L}$ mice. **(B, C)** Averaged mIPSCs halfwidth and decay time from the same neurons as in A. Data are presented as mean ± SEM, (n = 10 WT and n = 15 $\alpha3^{Q242L}$). All experiments are from female mice using the C57Bl/6N background. **(D)** Detailed information for measured electrophysiological parameters and statistical analyses.

Supplementary Methods. Detailed methods for electrocorticography electrode implantation surgery and recordings

Female WT (n = 7) and heterozygous $\alpha3^{Q242L}$ mice (n = 7) on C57Bl/6N background were used for ECoG experiments to study baseline epileptiform activity. ECoG electrode implantation was performed at > P40 as previously described (Bleakley *et al.*, 2021, DOI: 10.1093/brain/awab145). Mice were anaesthetised via isoflurane inhalation (4% for induction, 1–2% for maintenance throughout surgery), placed on a stereotaxic frame, and administered meloxicam (2.5 mg, subcutaneous) as an analgesic and lidocaine hydrochloride (10 mg, subcutaneous) to provide local anaesthesia to the scalp. An incision was made and three holes, each 1 mm in diameter, were drilled into the skull. Two of these holes were drilled bilaterally over the somatosensory cortex, with the third located immediately caudal to the lambdoid suture 0.5 mm lateral from the midline towards the right side of the skull. Stainless steel screw electrodes were implanted into each hole, with the anterior two screws implanted epidurally and used as the active channel electrodes, and the posterior screw implanted slightly more shallowly and used as the reference channel electrode. A ground electrode made of silver wire was affixed to the skull immediately caudal to the lambdoid suture 0.5 mm lateral from the midline towards the left side of the skull. The reference, ground, and two active channel electrodes were connected to a mouse electroencephalography (EEG) head mount (8201-EEG Pinnacle technology Inc.) via silver leads (Cat No. 785500, A-M Systems Inc.) and soldered in place. Self-curing acrylic resin was used to hold the head mount and electrodes in place. Mice were left to recover for at least one week prior to experimentation. ECoG data were sampled at 250 Hz and filtered (40 Hz low-pass, 0.5 Hz high-pass) using Sirenia Acquisition software (version 2.1.0, Pinnacle Technology Inc.). Twenty-four-hour recordings were conducted to characterize baseline EEG activity.

ECoG data were sampled at 250 Hz and filtered (40 Hz low-pass, 0.5 Hz high-pass) using Sirenia Acquisition software (version 2.1.0, Pinnacle Technology Inc.). Twenty-four-hour recordings were conducted to characterise baseline EEG activity.
